# Radiofrequency Ablation versus Hepatic Resection for Small Hepatocellular Carcinomas: A Meta-Analysis of Randomized and Nonrandomized Controlled Trials

**DOI:** 10.1371/journal.pone.0084484

**Published:** 2014-01-03

**Authors:** Yingqiang Wang, Qianqian Luo, Youping Li, Shaolin Deng, Shiyou Wei, Xianglian Li

**Affiliations:** 1 The Chinese Cochrane Centre, West China Hospital, Sichuan University, Chengdu, China; 2 Department of Medical Administration, 363 Hospital, Chengdu, China; 3 National Chengdu Center for Safety Evaluation of Drugs, West China Hospital, Chengdu, China; 4 West China Medical School, West China Hospital, Sichuan University, Chengdu, China; Icahn School of Medicine at Mount Sinai, United States of America

## Abstract

**Objectives:**

To evaluate the efficacy and safety of radiofrequency ablation (RFA) versus hepatic resection (HR) for early hepatocellular carcinoma (HCC) meeting the Milan criteria.

**Methods:**

A meta-analysis was conducted, and PubMed, Web of Science, the Cochrane Library, CBM, CNKI and VIP databases were systematically searched through November 2012 for randomized and nonrandomized controlled trials (RCTs and NRCTs). The Cochrane Collaboration's tool and modified MINORS score were applied to assess the quality of RCTs and NRCTs, respectively. The GRADE approach was employed to evaluate the strength of evidence.

**Results:**

Three RCTs and twenty-five NRCTs were included. Among 11,873 patients involved, 6,094 patients were treated with RFA, and 5,779 with HR. The pooled results of RCTs demonstrated no significant difference between groups for 1- and 3-year overall survival (OS), recurrence-free survival (RFS) and disease-free survival (DFS) (p>0.05). The 5-year OS (Relative Risk, RR 0.72, 95% CI 0.60 to 0.88) and RFS (RR 0.56, 95% CI 0.40 to 0.78) were lower with RFA than with HR. The 3- and 5-year recurrences with RFA were higher than with HR (RR 1.48, 95% CI 1.14 to 1.94, and RR 1.52, 95% CI 1.18 to 1.97, respectively), but 1-year recurrence and in-hospital mortality showed no significant differences between groups (p>0.05). The complication rate (RR 0.18, 95% CI 0.06 to 0.53) was lower and hospital stays (Mean difference -8.77, 95% CI −10.36 to −7.18) were shorter with RFA than with HR. The pooled results of NRCTs showed that the RFA group had lower 1-, 3- and 5-year OS, RFS and DFS, and higher recurrence than the HR group (p<0.05). But for patients with very early stage HCC, RFA was comparable to HR for OS and recurrence.

**Conclusion:**

The effectiveness of RFA is comparable to HR, with fewer complications but higher recurrence, especially for very early HCC patients.

## Introduction

Cancer is a major component of the global burden of disease (GBD). There were 2.49 billion disability-adjusted life years (DALYs), or 361 DALYs per 1000 population worldwide in 2010[1], and all neoplasms accounted for 7.6% (189 million DALYs) of global DALYs, an increase of 20 million DALYs (11.8%) compared with 2008[1], [2]. A study based on the human development index (HDI) of Bray F et al[3] estimated an increase in the incidence of new cancer cases of all kinds to 22.2 million annually by 2030, and that increases would be proportionally greatest in low-HDI settings compared with high-HDI countries (76% vs. 25%). There were 19.1 million DALYs for liver cancer in 2010, which accounted for 0.8% of the GBD or 10.1% of the DALYS for all neoplasms [1].

Hepatic resection (HR) and liver transplantation (LT) were recommended by the latest guidelines for early hepatocellular carcinoma meeting the Milan criteria, with the 5-year survival rate potentially reaching 50 to 75% [4], [5]. However, only 20–35% of patients are suitable for liver resection because of the low diagnosis rate for early HCC and to poor liver function [6]. In addition, few patients can be treated with liver transplantation because of the strict inclusion criteria, high cost, and limited donor liver resources.

Radiofrequency ablation (RFA) has higher efficacy and is associated with fewer complications and shorter hospital stays. RFA can also be administered repeatedly. Although RFA may gradually reach acceptability as an alternative treatment, the long-term efficacy and safety should still be evaluated systematically. Our previous study demonstrated that the overall quality of previously published systematic reviews and meta-analyses comparing RFA and HR for small hepatocellular carcinoma was poor, with an inadequate base of evidence[7]. Therefore, physicians may make an incorrect decision using these recommendations as best evidence without any additional quality evaluations to guide their clinical practice.

The purpose of this study was to retrieve the best available evidence and produce a meta-analysis comparing the long-term results of RFA and HR for early hepatocellular carcinoma to reduce research bias and improve the quality of evidence.

## Materials and Methods

### Inclusion criteria

The PICOS approach was used for eligibility criteria[8]:

**Population.** Patients met the Milan criteria (single HCC smaller than 5 cm in diameter or up to 3 nodules that were each smaller than 3 cm in diameter) or the UCSF criteria (single tumor smaller than 6.5 cm in diameter or up to 3 nodules that were each smaller than 4.5 cm in diameter and 8 cm in total diameter) with liver function Child-Pugh class A or B (the number of patients with Child-Pugh C was no more than 10%). Patients were without major vascular invasion and lymphatic spread or extrahepatic metastasis, and had no previous treatment of HCC with any anti-cancer treatment [Transcatheter Arterial Chemoembolization (TACE), Percutaneous Ethanol Injection (PEI), and Microwave Ablation (MWA)].

**Intervention.** RFA

**Comparison.** HR

**Outcome.** Efficacy: O_1_, overall survival rate (1-, 3-, 5-years), recurrence-free survival (RFS) (1-, 3-, 5-years), disease-free survival (DFS) (1-, 3-, 5-years).

Safety: O_2_, mortality; recurrence rate (1-, 3-, 5-years); complication rate

**Study design**. Randomized controlled trials (RCTs), non-randomized controlled trials (NRCTs), retrospective clinical or cohort study

DFS is defined as the time from randomization until recurrence of tumor or death form any cause[9]. In the case of HCC, the definition of DFS was identical as RFS where both recurrence and death form any causes are events[10]. However, the two terms were not clearly distinguished in the included RCTs or NRCTs, so we reported the two indicators respectively.

### Exclusion criteria

Conference abstracts, reviews, letters, systematic reviews or case reports were excluded. Metastatic liver cancer (i.e., colorectal liver metastases) or recurrent hepatocellular carcinoma after resection was excluded. Those studies that mixed other effective interventions in either treatment group or control group (i.e., TACE, PEI, LITT) as well as any that had a length of follow-up of less than one year were also excluded.

### Data sources and search strategy

We systematically searched 19 systematic reviews (SRs) comparing RFA with HR for small hepatocellular carcinoma in previously published studies and tracked the 39 primary studies included in these SRs. The six databases of PubMed, Web of Science, the Cochrane Library, CBM, CNKI, and VIP were systematically searched through November 2012. The following MeSH terms or free-words were used: (“hepatic resection[Title/Abstract]” OR “surgical resection[Title/Abstract]” OR “liver resection[Title/Abstract]” OR hepatectomy[MeSH Terms]) AND (radiofrequency[Title/Abstract] OR “radiofrequency ablation[Title/Abstract]” OR “catheter ablation[MeSH Terms]” OR “RFA[Title/Abstract]”) AND (“hepatocellular carcinoma[Title/Abstract]” OR “liver neoplasm[MeSH Terms]” OR “liver tumor[Title/Abstract]” OR “liver cancer[Title/Abstract]”). We did not restrict the language of publication.

### Review selection and data extraction

The PRISMA statement was followed when searching and screening the literature[8]. Two reviewers (WYQ, LQQ) selected articles independently by browsing titles and abstracts according to predefined inclusion and exclusion criteria. If necessary, judgment was made by viewing the full text. The two reviewers extracted data using standardized forms independently if the publication met the inclusion criteria. Discrepancies between the two reviewers were resolved by discussion or by a third person (LYP).The extracted contents included first author, publication year, type of study, inclusion criteria, number of participants, age, gender, number of nodules, tumor size, the Child-Pugh score, length of follow-up, and clinical outcomes.

### Quality assessment

Two reviewers (WYQ, LQQ) evaluated the methodological quality of included studies independently. The tool for evaluating the risk of bias in the Cochrane Handbook for Systematic Reviews of Interventions[11] and the modified MINORS scores questionnaire[12] were applied to assess the quality of RCTs and NRCTs, respectively. GRADE profiler 3.6 was employed to evaluate the strength of the evidence.

### Statistical analysis

Meta-analysis was conducted using RevMan 5.1 software. For dichotomous variables, the relative risk (RR) and the odds ratio (OR) with 95% confidence intervals (CIs) were used for RCTs and NRCTs, respectively, For continuous variables, mean difference (MD) with 95% CI was applied. The fixed-effect model (Mantel-Haenszel) was used if the result of the heterogeneity test was p>0.05 and if I^2^<50%. Otherwise, the random-effect model was applied. A p value <0.05 was considered significant.

### Subgroup analysis

Subgroup analysis was applied based on predetermined subgroup factors (i.e., tumor size, number of nodules, and Child-Pugh class).

### Sensitivity analysis

We excluded the studies that might cause heterogeneity and re-estimated the combined effect of values, and then compared the results with the primary outcomes to verify the robustness of the outcomes.

### Publication bias

The funnel plot and Egger's test were applied by Revman 5.1 and Stata 10.0 software, respectively. If the result of the test was p<0.05, it suggested that potential publication bias may exist. Otherwise, publication bias was considered absent.

## Results

### Search results

**Figure 1:**
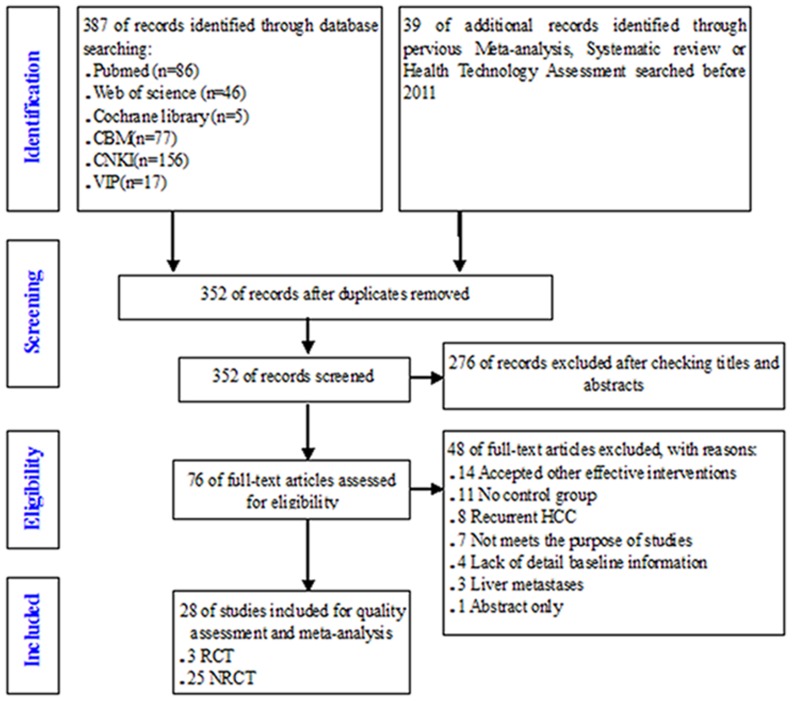
PRISMA flowchart of searching and selecting guidelines.

**Figure 2:**
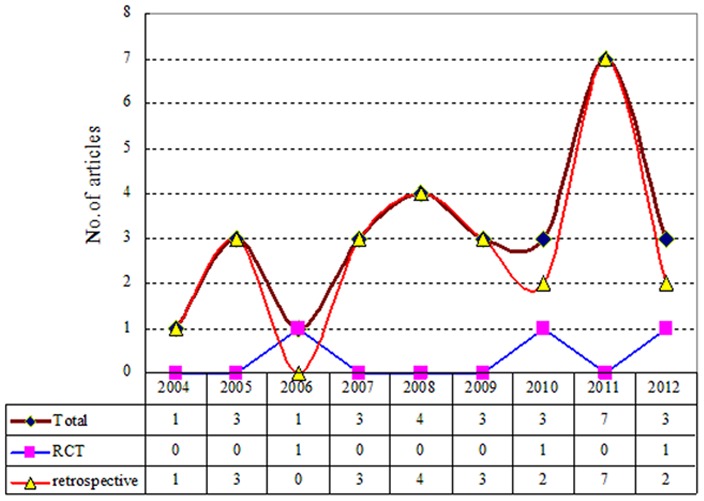
Bibliometric map of included studies.

After initial screening, 76 studies were identified. Of these, 48 studies were removed after viewing full-texts for various reasons: a) mixed with other effective interventions (i.e., TACE, PEI) in either intervention group or control group (14 articles), b) without control group (11 articles), c) recurrent HCC (8 articles), d) did not meet the purpose of this study for other reasons (7 articles), e) lack of detailed baseline information (4 articles), f) metastatic liver cancer (3 articles), and g) abstract only (1 article). Finally, 28 studies including 3 RCTs [10], [13], [14] and 25 NRCTs [6], [15]–[38] published between 2004 and 2012 were included (see Figure 1, Figure 2).

### Baseline characteristics of included studies

A total of 11,873 patients with primary HCC were included in the 28 studies. Among those, 8,567 cases (72%) were males, and 6,094 cases were treated with RFA and 5,779 cases with hepatic resection. There were 11,251 cases (94.8%) with Child-Pugh class A, 1,632 cases (13.7%) with Child-Pugh class B, and only 17 cases (0.1%) with Child-Pugh class C. Patients had an average age of 50 years, with varying degrees of HBsAg-positivity and cirrhosis. The mean length of follow-up ranged from 10 to 60 months (Table 1).

**Table 1:** Baseline characteristics of RCTs and NRCTs.

Author, year	Study types	Included criteria	Group	Pts	Men %	Age, year (Mean±SD)	Solitary/Multiple	HBsAg(+), %	Cirrhosis, %	Child-Pugh (A/B/C)	Tumor size (Mean±SD)	Follow-up(mo) (Mean±SD)	Quality*
Feng K,2012[13]	RCT	HCC tumor≤4 cm,nodels ≤2,Child A/B	RFA	84	94.0	51.0(24–83)‡	48/36	_	71.4	39/45/0	2.4±0.6	36.0#	B
			HR	84	89.3	47.0(18–76)‡	52/32	_	76.2	43/41/0	2.6±0.8	36.0^#^	
Huang JW,2010[10]	RCT	Milan criteria, Child A/B	RFA	115	68.7	56.6±14.3	84/31	87.8	58.3	110/5/0	≤3 cm(57),>3 cm(27)^£^	3.1(0.5–5)‡	B
			HR	115	73.9	55.9±12.7	89/26	90.4	65.2	106/9/0	≤3 cm(45),>3 cm(44)^£^	3.87(0.1–5)‡	
Chen MS,2006[14]	RCT	Solitary HCC≤5 cm,Child A	RFA	71	78.9	51.9±11.2	71/0	_	_	71/0/0	≤3 cm(37);3.1–5 cm(34)^£^	27.9±10.6	B
			HR	90	83.3	49.4±10.9	90/0	_	_	90/0/0	≤3 cm(42);3.1–5 cm(48)^£^	29.2±11.9	
Wang JH,2012[15]	Retro-	BCLC very early stage HCC	RFA	52	67.3	≤60(29)	52/0	61.5	_	52/0/0	_	2.5(1.4–4.1)‡	18
			HR	52	73.1	≤60(35)^£^	52/0	65.4	_	52/0/0	_	2.3(1.5–3.7)‡	
		BCLC early stage HCC	RFA	254	63.4	≤60(85)^£^	173/81	36.6	_	191/63/0	≤2 cm(60)^£^	2.4(1.5–3.6)‡	
			HR	208	80.8	≤60(113)^£^	189/19	54.3	_	205/3/0	≤2 cm(6)^£^	1.4(1.4–3.4)‡	
Peng ZW,2012[16]	Retro-	Solitary HCC tumor ≤2 cm,Child A	RFA	71	88.7	53.1±12.1	71/0	97.2	95.8	58/0/0	1.2±0.6	59.0±23.2	18
			HR	74	87.8	51.5±12.1	74/0	94.6	83.8	62/0/0	1.1±0.5	57.5±20.0	
Kong WT,2011[19]	Pro-	HCC tumor≤5 cm,and nodules≤3,Child A/B	RFA	47	78.7	57.0±14.0	40/7	83.0	72.3	40/7/0	<2 cm(15),2∼5 cm(40)^£^	6–69^#^	18
			HR	40	87.5	53.0±13.0	38/2	65.0	75.0	37/3/0	<2 cm(9),2∼5 cm(34)^£^	6–69^#^	
Hung HH,2011[22]	Retro-	HCC tumor≤5 cm,and nodules≤3;Child A/B	RFA	190	63.7	67.4±11.5	152/38	46.3	_	_/_/_	2.4±0.9	42.1±23.5	17
			HR	229	80.3	60.1±12.6	181/48	59.8	_	_/_/_	2.9±1.1	42.1±23.5	
Cho CM,2005[37]	Retro-	HCC tumor≤3 cm, and nodels≤3;Child A	RFA	99	76.8	58.0¶	_/_	69.7	_	99/0/0	3.1±0.8	23.0±9.4	17
			HR	61	78.7	57.0¶	_/_	82.0	_	61/0/0	3.4±1.0	21.9±9.8	
Fu J,2011[18]	Retro-	Solitary≤5 cm, or tumor≤4,and each≤3 cm,Child A/B, aged>60	RFA	76	77.6	69.2±8.7	69/7	72.4	69.7	64/12/0	<3 cm(34),3∼5 cm(42)^£^	_	16
			HR	52	76.9	70.5±9.3	46/5	86.5	71.2	46/5/0	<3 cm(23),3∼5 cm(28)^£^	_	
Nishikawa H,2011[20]	Retro-	Solitary HCC≤3 cm	RFA	162	58.6	67.4±9.7	162/0	5.6	_	102/22/3	2.0±0.6	3.1(0.2–7)‡	16
			HR	69	72.5	68.4±8.7	69/0	11.6	_	45/5/0	2.7±0.5	3.3(0.7–7)‡	
Ikeda K,2011[21]	Retro-	Small HCC≤3 cm;Child A/B	RFA	236	61.4	67.0(38–87)‡	195/41	10.2	_	_/_/_	1.8(0.8–3)‡	3.7(0.1–9.9)‡	16
			HR	138	73.2	62.5(29–80)‡	114/24	33.3	_	_/_/_	2.0(0.5–3)‡	4.5(0.1–10.0)‡	
Huang JW,2011[23]	Retro-	HCC tumor≤5 cm,and nodules≤3;Child A;	RFA	413	87.4	54.67±12.18	313/100	94.7	100.0	413/0/0	4.0±1.2	36.1±12.4	16
			HR	648	75.5	46.13±16.89	507/141	92.3	100.0	648/0/0	3.6±1.5	33.7±17.4	
Guo WX,2010[24]	NRCT	HCC tumor≤5 cm, and 2 or 3 nodules. Child A/B	RFA	86	73.3	52.5(26–80)‡	0/86	91.9	86.0	84/2/0	3.2(1.5–5)‡	27.0(9–72)‡	16
			HR	73	78.1	50.0(17–68)‡	0/73	97.3	91.8	71/2/0	3.5(1.7–5)‡	30.0(7–84)‡	
Ueno S,2009[26]	Retro-	Milan criteria,	RFA	155	64.5	66.0(40–79)‡	101/54	16.1	_	52/92/11	2.0±0.1	35.0±1.7	16
			HR	123	66.7	67.0(28–85)‡	110/13	17.9	_	91/31/1	2.7±0.1	36.8±1.5	
Santambrogio R,2009[27]	Retro-	Solitary tumor≤5 cm,Child A	RFA	74	79.7	68.0±7.0	59/15	16.2	100.0	74/0/0	2.6±1.1	38.2±28.4	16
			HR	78	70.5	68.0±8.0	68/6	12.8	100.0	78/0/0	2.9±1.2	36.2±23.5	
Kobayashi M,2009[6]	Retro-	HCC tumor≤3 cm,and nodels≤3;Child A	RFA	209	65.6	67.0(38–87)‡	169/40	11.0	_	209/0/0	1.8(0.8–3)‡	3.3(0.1–12.2)‡	16
			HR	199	73.4	62.0(29–80)‡	168/31	31.7	_	199/0/0	2.0(0.9–3)‡	3.3(0.1–12.2)‡	
Zhou T,2007[32]	Retro-	HCC tumor≤5 cm,and nodels≤3;Child A/B	RFA	47	78.7	57.0±14.0	40/7	83.0	72.3	40/7/0	≤2 cm(8);2–5 cm(39)^£^	_	16
			HR	40	87.5	53.0±13.0	38/2	65.0	75.0	37/3/0	≤2 cm(7);2–5 cm(33)^£^	_	
Lupo L,2007[34]	Retro-	Single nodule range 3–5 cm;Child A/B	RFA	60	78.3	68.0(42–85)‡	60/0	20.0	100.0	44/16/0	3.7(3–5)‡	27.0±18.7	16
			HR	42	78.6	67.0(28–80)‡	42/0	23.8	100.0	28/14/0	4.0(3–5)‡	31.3±24.3	
Bu XY,2010[25]	Retro-	HCC tumor≤5 cm;Child A/B	RFA	46	87.0	55.9±7.4	38/8	_	84.8	25/21/0	≤3 cm(20),3–5 cm(26)^£^	36.2(9–67)‡	15
			HR	42	85.7	53.9±10.7	38/4	_	90.5	36/6/0	≤3 cm(14),3–5 cm(28)^£^	32.3(6–72)‡	
Liu H,2011[17]	Retro-	Solitary HCC≤5 cm;Child A	RFA	32	81.3	46.1±24.1	32/0	_	_	32/0/0	<3 cm(14),3∼5 cm(18)^£^	_	15
			HR	35	82.9	48.2±15.6	35/0	_	_	35/0/0	<3 cm(12),3∼5 cm(19)^£^	_	
Hiraoka A,2008[28]	Retro-	Solitary HCC≤3 cm;Child A/B	RFA	105	72.4	69.4±9.1	105/0	_	_	79/26/0	2.0±0.5	847.4±700.3†	15
			HR	59	74.6	62.4±10.6	59/0	_	_	54/5/0	2.3±0.6	927.1±698.4†	
Hasegawa K,2008[29]	Retro-	HCC tumor≤3 cm,and nodels≤3;Child A/B	RFA	3022	64.1	69.0(52–80)‡	2189/833	_	_	2288/734/0	2.0(1–3)‡	10.4(4.8–16.7)‡	15
			HR	2857	74.0	67.0(48–77)‡	2410/447	_	_	2570/287/0	2.2(1.2–3)‡	10.4(4.8–16.7)‡	
Guglielmi A,2008[30]	Retro-	HCC ≤6 cm,and single or multiple tumor≤3; Child A/B	RFA	109	80.7	≤65(38)^£^ >65(65)^£^	65/44	12.8	_	64/45/0	≤3 cm(32),3–6 cm(77)^£^	23.0(3–92)‡	15
			HR	91	80.2	≤65(47)^£^ >65(44)^£^	69/22	11.0	_	69/22/0	≤3 cm(31),3–6 cm(66)^£^	32.0(3–120)‡	
Montorsi M,2005[35]	Pro-	Solitary HCC ≤5 cm;Child A/B	RFA	58	74.1	67.0±6.0	58/0	_	100.0	40/18/0	_	25.7±17.5	15
			HR	40	82.5	67.0±9.0	40/0	_	100.0	32/8/0	_	22.4±16.7	
Hong SN,2005[36]	Retro-	Solitary HCC≤4 cm, Child-Pugh score 5 or those without cirrhosis	RFA	55	74.5	59.1±9.6	55/0	72.7	87.3	55/0/0	2.4±0.6	22.7(15–57)‡	15
			HR	93	74.2	49.2±9.9	93/0	87.1	80.6	93/0/0	2.5±0.8	25.5(5–57)‡	
Vivarelli M,2004[38]	Retro-	HCC tumor ≤3 cm, cirrhosis	RFA	79	84.8	67.8±8.7	46/33	16.5	84.8	43/36/0	≤3 cm(22);>3 cm(57)^£^	15.6±11.7	15
			HR	79	72.2	65.2±8.2	66/13	25.3	72.2	70/9/0	≤3 cm(21);>3 cm(58)^£^	28.9±17.9	
Abu-Hilal M,2008[31]	Retro-	Milan criteria	RFA	34	79.4	65.0	34/0	_	_	27/7/0	3.0(2–5)‡	30.0(0–60)‡	14
			HR	34	76.5	67.0	34/0	_	_	25/9/0	3.8(1.3–5)‡	43.0(2–129)‡	
Gao W,2007[33]	Retro-	Small HCC≤3 cm	RFA	53	77.4	57.1(31–81)‡	29/24	_	_	40/11/2	2.5±0.4	28.4(4–70)‡	14
			HR	34	82.4	51.5(38–67)‡	32/2	_	_	33/1/0	2.6±0.4	25.2(5–66)‡	

*The quality of RCTs was assessed by Cochrane handbook for intervention of systematic, which these of NRCTs were scored by MINORS checklist (Total score of 18);

: Mean;

: Median (range);

: Mean days of follow-up;

#:Intervals or range of follow-up;

:Tumor size or Age (No. of patient)

Blinding was not performed in the three RCTs, and the overall quality of evidence level was grade B [10], [13], [14]. The quality of the NRCTs was moderate, with an estimated mean MINORS score (18 of total) of 15.8 (95% CI, 15.4–16.3). Only 15 (60%) studies were scored ≥16 [6], [15]–[38] (Table 1).

### Clinical outcome of HCC patients with tumor size smaller than 5 cm

#### Overall Survival

**Figure 3:**
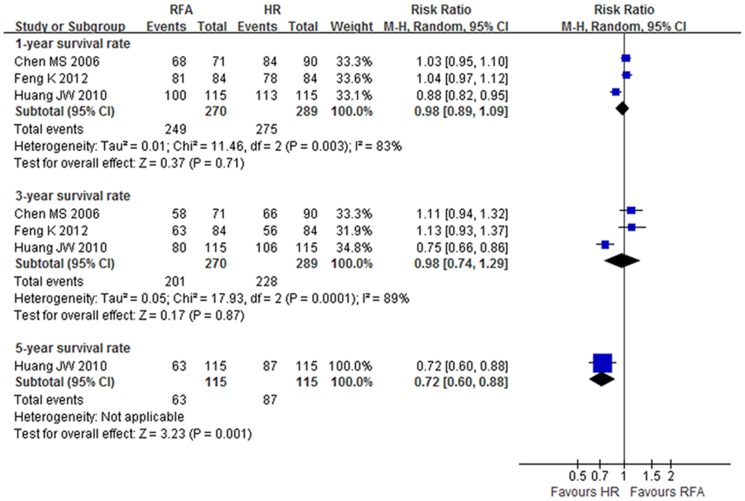
Overall survival (OS) at 1-, 3- and 5-year in RCTs.

The pooled meta-analysis from the three RCTs [10], [13], [14] showed no significant difference of 1- and 3-year survival rates between groups (RR 0.98, 95% CI: 0.89–1.09, NNH = 33.3, p = 0.71; and RR 0.98, 95% CI: 0.74–1.29, NNH = 22.2, p = 0.87, respectively) (level of evidence: moderate). Only Huang et al[10] demonstrated that the 5-year survival rate in the RFA group was lower than in the HR group (RR 0.75, 95% CI: 0.60–0.88, NNH = 4.8, p = 0.001) (level of evidence: high) (Figure 3, Table 2).

**Table 2:** Summary of finding table for HCC patients with tumor less than 5

			Event,%		Effect estimate^#^				Illustrative comparative risks(95%CI)^Ψ^		
Indicators	Years	No. of Participants (studies)	RFA	HR	OR,95% CI	I^2^ (%)	P value	NNT or NNH	Assumed risk HR(control)(per 1000)	Corresponding risk RFA(per 1000)	Quality of evidence (GRADE)
Overall survival	1-y	559(3)	92.2	95.2	0.98[0.89,1.09]	83	0.71	33.3†	**933**	**914** (830 to 1000)	⊕⊕⊕^⊖^ Moderate
	3-y	559(3)	74.4	78.9	0.98[0.74,1.29]	89	0.87	22.2†	**733**	**718** (542 to 946)	⊕⊕⊕^⊖^ Moderate
	5-y	230(1)	54.8	75.7	0.72[0.60,0.88]	—	—	4.8†	**757**	**545** (454 to 666)	⊕⊕⊕⊕High
Recurrence-free survival	1-y	398(2)	85.4	85.4	1.01[0.92,1.11]	29	0.88	_	**855**	**864** (787 to 949)	⊕⊕⊕⊕High
	3-y	398(2)	52.3	56.3	0.95[0.60,1.52]	84	0.85	25.0†	**554**	**526** (332 to 842)	⊕⊕⊕^⊖^ Moderate
	5-y	230(1)	28.7	51.3	0.56[0.40,0.78]	—	—	4.4†	**513**	**287** (205 to 400)	⊕⊕⊕⊕High
Disease-free survival	1-y	180(1)	91.1	86.7	1.05[0.95,1.17]^&^	—	0.34	22.7^‡^	**867**	**910** (824 to 1000)	⊕⊕⊕^⊖^Moderate
	2-y	180(1)	68.9	76.7	0.90[0.75,1.08]^&^	—	0.24	12.8†	**767**	**690** (575 to 828)	⊕⊕⊕^⊖^Moderate
	3-y	180(1)	60.0	68.9	0.87[0.70,1.08]^&^	—	0.22	11.2†	**689**	**599** (482 to 744)	⊕⊕⊕^⊖^Moderate
	4-y	180(1)	47.8	51.1	0.93[0.70,1.26]^&^	—	0.65	30.3†	**511**	**475** (358 to 644)	⊕⊕⊕^⊖^Moderate
Recurrence	1-y	398(2)	15.6	11.1	1.41[0.85,2.35]^&^	0	0.19	22.2†	**109**	**154** (93 to 256)	⊕⊕⊕^⊖^ Moderate
	3-y	398(2)	43.2	29.1	1.48[1.14,1.94]^&^	0	0.004*	7.1†	**304**	**450** (347 to 590)	⊕⊕⊕^⊖^ Moderate
	5-y	230(1)	63.5	41.7	1.52[1.18,1.97]^&^	—	0.001*	4.6†	**417**	**634** (492 to 821)	⊕⊕⊕^⊖^ Moderate
Local recurrence		398(2)	20.6	14.1	1.46[0.97,2.21]^&^	0	0.07	15.4†	**159**	**232** (154 to 351)	⊕⊕⊕^⊖^ Moderate
Distant recurrence		398(2)	5.0	4.0	1.25[0.50,3.10]^&^	0	0.63	100.0†	**40**	**50** (20 to 124)	⊕⊕⊕^⊖^ Moderate
In-hospital Mortality		559(3)	0.0	0.3	0.42[0.02,10.19]^&^	—	0.59	333.3^‡^	**0**	**0** (0 to 0)	⊕⊕⊕^⊖^ Moderate
Complication rate		559(3)	5.9	34.6	0.18[0.06,0.53]	74	0.002*	3.5^‡^	**278**	**50** (17 to 147)	⊕⊕⊕^⊖^ Moderate
Hospital stay		559(3)	—	—	-8.77[−10.36,−7.18]^△^	83	<0.001*	_	_	RFA group was 8.77 lower(10.36 to 7.18 lower)	⊕⊕⊕^⊖^ Moderate

Ψ: The basis for the assumed risk (e.g. the median control group risk across studies) is provided in footnotes. The corresponding risk (and its 95% confidence interval) is based on the assumed risk in the comparison group and the relative effect of the intervention (and its 95%CI);

#, random-effect model;

&, fixed-effect model;

△: Mean Difference (IV, Random, 95% CI).

,NNH(Number needed to harm);

‡,NNT(Number needed to treat);

*:p<0.05

The pooled meta-analysis from the NRCTs showed that the 1-, 3- and 5-year survival rates in the RFA group were significantly lower than in the HR group (OR 0.78, 95% CI: 0.63–0.97, NNH = 166.7; OR 0.67, 95% CI: 0.52–0.85, NNH = 12.5; and OR 0.58, 95% CI: 0.36–0.94, NNH = 10.6, respectively) (level of evidence: very low) (Table 3).

**Table 3:** Summary of finding table for HCC patients with tumor size less than 5

				Event,%		Effect estimate^#^				Illustrative comparative risks(95%CI)^Ψ^		
Indicators	subgroup	Years	No. of Participants (studies)	RFA	HR	(OR,95% CI)	I^2^ (%)	P value	NNT or NNH	Assumed risk HR(Control)(Per 1000)	Corresponding risk RFA(Per 1000)	Quality of evidence (GRADE)
Overall Survival	All	1-y	10621(23)	96.2	96.8	0.78[0.63,0.97]	35	0.02*	166.7†	**941**	**926** (909 to 939)	⊕^⊖^^⊖^^⊖^ **very low**
		3-y	5115(23)	75.4	83.4	0.67[0.52,0.85]^&^	57	0.0008*	12.5†	**774**	**696** (640 to 744)	⊕^⊖^^⊖^^⊖^ **very low**
		5-y	4278(15)	63.7	73.1	0.68[0.48,0.97]^&^	82	0.03*	10.6†	**731**	**649** (566 to 725)	⊕^⊖^^⊖^^⊖^ **very low**
	Child A	1-y	2460(11)	93.2	95.4	0.58[0.36,0.94]	42	0.002*	45.5†	**979**	**964** (950 to 975)	⊕^⊖^^⊖^^⊖^ **very low**
		3-y	2527(12)	74.9	84.7	0.58[0.36,0.94]^&^	71	0.03*	10.2†	**844**	**758** (661 to 836)	⊕^⊖^^⊖^^⊖^ **very low**
		5-y	2076(7)	63.1	76.7	0.59[0.36,0.99]^&^	75	0.05*	7.4†	**789**	**688** (574 to 787)	⊕^⊖^^⊖^^⊖^ **very low**
Recurrence-free survival	All	1-y	2388(8)	75.8	79.7	0.78[0.64,0.95]	0	0.01*	25.6†	**782**	**737** (697 to 773)	⊕^⊖^^⊖^^⊖^ **very low**
		3-y	2388(8)	41.9	53.6	0.67[0.56,0.79]	38	<0.001*	8.5†	**528**	**428** (385 to 469)	⊕^⊖^^⊖^^⊖^ **very low**
		5-y	1845(4)	27.8	41.1	0.63[0.40,1.00]^&^	73	0.05*	7.5†	**398**	**294** (209 to 398)	⊕^⊖^^⊖^^⊖^ **very low**
	Child A	1-y	1922(5)	75.9	79.6	0.80[0.64,1.00]	0	0.05*	27.0†	**763**	**720** (673 to 763)	⊕^⊖^^⊖^^⊖^ **very low**
		3-y	1922(5)	43.7	54.6	0.67[0.56,0.81]	29	<0.001*	9.2†	**548**	**448** (404 to 495)	⊕^⊖^^⊖^^⊖^ **very low**
		5-y	1614(3)	30.2	42.2	0.64[0.35,1.17]^&^	82	0.15	8.3†	**429**	**325** (208 to 468)	⊕^⊖^^⊖^^⊖^ **very low**
Disease-free survival	All	1-y	2766(12)	70.1	83.9	0.46[0.38,0.55]	46	<0.001*	7.2†	**830**	**692** (650 to 729)	⊕⊕^⊖^^⊖^ **low**
		3-y	2698(11)	37.6	57.3	0.49[0.34,0.69]^&^	67	<0.001*	5.1†	**559**	**383** (301 to 467)	⊕^⊖^^⊖^^⊖^ **very low**
		5-y	2549(9)	21.7	38.6	0.52[0.32,0.84]^&^	74	0.007*	5.9†	**286**	**172** (114 to 252)	⊕^⊖^^⊖^^⊖^ **very low**
	Child A	1-y	1310(4)	78.7	87.9	0.51[0.38,0.69]	45	<0.001*	10.9†	**886**	**799** (747 to 843)	⊕⊕^⊖^^⊖^ **low**
		3-y	1310(4)	47.0	63.9	0.50[0.40,0.63]	0	<0.001*	5.9†	**627**	**457** (402 to 514)	⊕⊕⊕^⊖^ **Moderate**
		5-y	1165(2)	32.5	43.4	0.62[0.49,0.80]	4	0.0002*	9.2†	**420**	**310** (262 to 367)	⊕⊕^⊖^^⊖^ **low**
Recurrence	All	1-y	7019(6)	25.2	16.9	1.50[1.03,2.19]^&^	61	0.03*	12.4†	**171**	**260** (237 to 283)	⊕^⊖^^⊖^^⊖^ **very low**
		3-y	1140(5)	57.1	41.4	1.87[1.23,2.84]^&^	60	0.004*	6.4†	**406**	**585** (524 to 642)	⊕^⊖^^⊖^^⊖^ **very low**
		5-y	945(3)	75.0	58.0	2.34[1.76,3.11]	0	<0.001*	5.9†	**590**	**771** (717 to 817)	⊕⊕^⊖^^⊖^ **low**
	Child A	1-y	335(3)	21.5	19.0	1.18[0.69,2.03]	0	0.54	40.0†	**180**	**206** (132 to 308)	⊕^⊖^^⊖^^⊖^ **very low**
		3-y	335(3)	50.6	46.6	1.06[0.38,3.00]^&^	81	0.91	25.0†	**423**	**437** (218 to 687)	⊕^⊖^^⊖^^⊖^ **very low**
		5-y	268(2)	68.6	67.2	1.04[0.18,6.04]^&^	90	0.96	71.4†	**684**	**692** (280 to 929)	⊕^⊖^^⊖^^⊖^ **very low**
In-hospital Mortality		—	3061(14)	0.1	0.3	0.47[0.13,1.76]	0	0.26	500.0^‡^	**0**	**0** (0 to 0)	⊕⊕^⊖^^⊖^ **low**
Complication rate	All		3073(15)	8.3	17.7	0.37[0.29,0.47]	34	<0.001*	10.6^‡^	**192**	**81** (64 to 100)	⊕⊕^⊖^^⊖^ **low**
	Child A		1585(5)	7.7	16.7	0.35[0.25,0.50]	0	<0.001*	11.1^‡^	**314**	**138** (103 to 186)	⊕⊕^⊖^^⊖^ **low**
Hospital stay		—	1565(4)	—	—	-6.74[−11.33,−2.14]^△^	98	0.004*	-	-	RFA was **6.74 lower** (2.14 to11.33)	⊕^⊖^^⊖^^⊖^ **very low**

Ψ: The basis for the assumed risk (e.g. the median control group risk across studies) is provided in footnotes. The corresponding risk (and its 95% confidence interval) is based on the assumed risk in the comparison group and the relative effect of the intervention (and its 95%CI);

#, random-effect model; &, fixed-effect model;

△: Mean Difference (IV, Random, 95% CI).

,NNH(Number needed to harm);‡,NNT(Number needed to treat);

*:p<0.05

#### Recurrence-free survival

**Figure 4:**
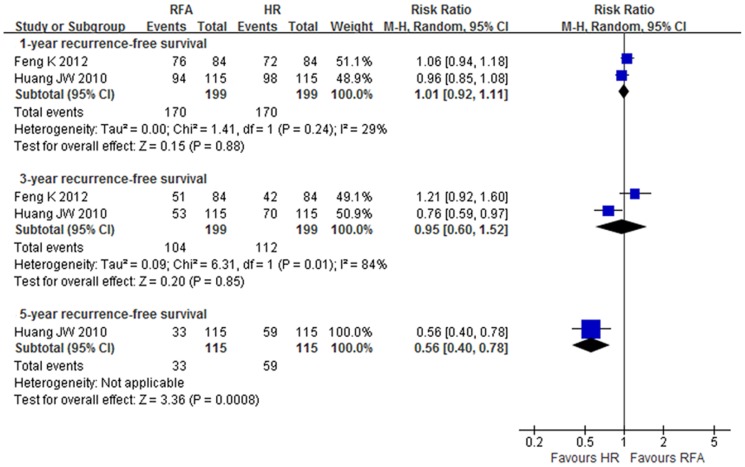
Recurrence-free survival rate (RFS) at 1-, 3- and 5-year in RCTs.

The meta-analysis from the RCTs showed no significant difference of 1- and 3-year recurrence-free survival rates between groups (level of evidence: moderate to high). The 5-year survival rate in the RFA group, however, was lower than in the HR group (RR 0.56, 95% CI: 0.40 to 0.78, NNH = 4.4) (level of evidence: high) (Figure 4, Table 2).

The pooled results from the NRCTs demonstrated that the 1-, 3- and 5-year recurrence-free survival rates in the RFA group were lower than in the HR group (OR 0.78, 95% CI: 0.64–0.95, NNH = 25.6; OR 0.67, 95% CI: 0.56–0.79, NNH = 8.5; and OR 0.63 95% CI: 0.40–1.00, NNH = 7.5, respectively) (level of evidence: very low). However, there was no significant difference between groups of 5-year recurrence-free survival rates for patients with Child-Pugh class A (OR 0.64, 95% CI: 0.35–1.17; NNH = 8.3) (level of evidence: very low) (Table 3).

#### Disease-free survival

Only one RCT [14] reported the disease-free survival rate, showing no significant difference between groups (level of evidence: moderate).

The pooled meta-analysis from the NRCTs showed that the 1-, 3- and 5-year disease-free survival rates in the RFA group were significantly lower than in the HR group (OR 0.46, 95% CI: 0.38–0.55, NNH = 7.2; OR 0.49, 95% CI: 0.34–0.69, NNH = 5.1; and OR 0.52, 95% CI: 0.32–0.84, NNH = 5.9, respectively) (level of evidence: very low to low) (Table 3).

#### Recurrence

**Figure 5:**
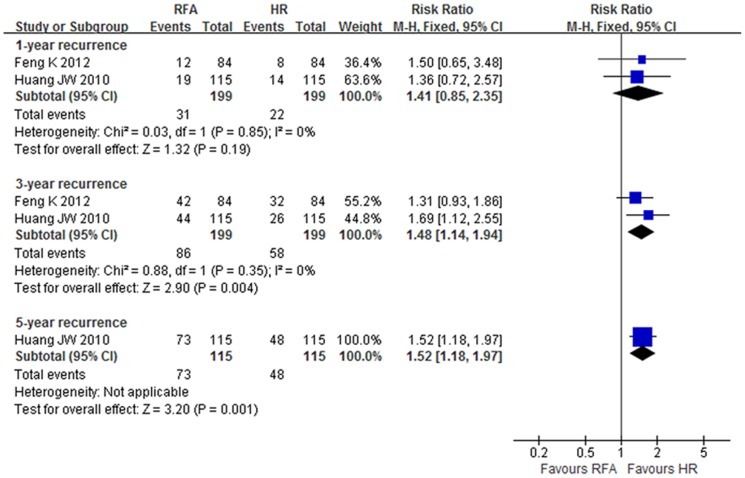
Recurrence rate at 1-, 3- and 5-year in RCTs.

The pooled results from the RCTs showed that the 3- and 5-year recurrence rates in the RFA group were significantly higher than in the HR group (RR 1.48, 95% CI: 1.14–1.94, NNH = 7.1; and RR 1.52, 95% CI: 1.18–1.97, NNH = 4.6, respectively) (level of evidence: moderate). However, there were no significant differences of 1-year recurrence rates or of local or distant recurrence rates between groups (level of evidence: moderate) (Figure 5, Table 2).

The results of meta-analysis for the NRCTs showed that the 1-, 3- and 5-year recurrence rates in the RFA group were higher than in the HR group (OR 1.5, 95% CI: 1.03–2.19, NNH = 12.4; OR 1.87, 95% CI: 1.23–2.84, NNH = 6.4; and OR 2.34, 95% CI: 1.76–3.11, NNH = 5.9, respectively) (level of evidence: very low to low). However, there were no significant differences between groups for patients with Child-Pugh class A (level of evidence: very low) (Table 3).

#### In-hospital mortality, complication rate and length of hospital stay

**Figure 6:**
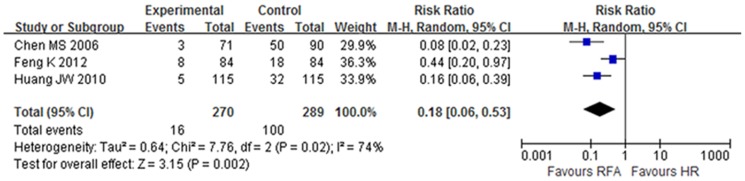
Complication rate of RCTs.

The pooled results of the RCTs showed no significant differences of in-hospital mortality between groups, but the complication rate in the RFA group was lower than in the HR group (RR 0.18, 95% CI: 0.06–0.53, NNT = 3.5) (level of evidence: moderate). Length of hospital stay in the RFA group was 8.77 days fewer than in the HR group (95% CI: 10.36 to 7.18 lower) (level of evidence: moderate) (Figure 6, Table 2).

The pooled results of the NRCTs for in-hospital mortality and complication rate were similar to those of the RCTs, but the hospital length of stay in the RFA group was 6.74 days fewer than in the HR group (95% CI: 11.33 to 2.14 lower) (level of evidence: very low) (Table 3).

### Clinical outcome of HCC patients with tumor size between 3 and 5 cm

The pooled results of the RCTs showed no significant difference of overall survival rates between the groups for single HCC patients with tumor size ranging from 3 to 5 cm in diameter (level of evidence: low to moderate) (Table 4).

**Table 4:** Summary of finding table for Solitary HCC with tumor size≤3 cm and 3–5 cm in RCTs.

				Event,%		Effect estimate^#^				Illustrative comparative risks(95%CI)^Ψ^		
Indicators	Subgroup	Years	No. of Participants (studies)	RFA	HR	OR,95% CI	I^2^ (%)	P value	NNH	Assumed risk HR(control)(per 1000)	Corresponding risk RFA(per 1000)	Quality of evidence (GRADE)
Overall survival	Tumor size≤3 cm	1-y	181(2)	93.6	97.7	0.49[0.03,8.45]	56	0.62	24.4	**976**	**952** (550 to 997)	⊕⊕^⊖^^⊖^ **low**
		3-y	181(2)	74.5	81.6	0.65[0.32,1.33]^&^	49	0.24	14.1	**814**	**743** (512 to 891)	⊕⊕⊕^⊖^ **Moderate**
		4-y	181(2)	74.5	78.2	0.80[0.20,3.22]	73	0.76	27.0	**779**	**738** (413 to 919)	⊕⊕^⊖^^⊖^ **low**
	Tumor size 3∼5 cm	1-y	153(2)	91.8	96.7	0.38[0.03,4.85]	54	0.46	20.4	**969**	**928** (775 to 981)	⊕⊕^⊖^^⊖^ **low**
		3-y	153(2)	62.3	83.7	0.26[0.05,1.36]	69	0.11	4.7	**842**	**637** (444 to791)	⊕⊕^⊖^^⊖^ **low**
		4-y	153(2)	57.4	72.8	0.51[0.25,1.03]^&^	3	0.06	6.5	**733**	**583** (407 to737)	⊕⊕⊕^⊖^ **Moderate**

Ψ: The basis for the assumed risk (e.g. the median control group risk across studies) is provided in footnotes. The corresponding risk (and its 95% confidence interval) is based on the assumed risk in the comparison group and the relative effect of the intervention (and its 95%CI);

# Statistical method (M-H, random-effect model).

*:p<0.05

The results from the NRCTs showed that the 5-year survival rate in the RFA group was lower than in the HR group (OR 0.43, 95% CI: 0.25–0.73, NNH = 5.3), but there were no significant differences of 1-and 3-year survival rates between groups (level of evidence: very low). The 1-, 3- and 5-year disease-free survival rates in the RFA group were lower than in the HR group (OR 0.47, 95% CI: 0.26–0.83, NNH = 6.9; OR 0.35, 95% CI: 0.18–0.67, NNH = 6.1; and OR 0.18, 95% CI: 0.05–0.61, NNH = 9.9, respectively) (level of evidence: very low to low) (Table 5).

**Table 5:** Summary of finding table for HCC patients with tumor size between 3 and 5

			Event,%		Effect estimate^#^				Illustrative comparative risks(95%CI)^Ψ^		
Indicators	Years	No. of Participants (studies)	RFA	HR	OR,95% CI	I^2^ (%)	P value	NNT or NNH	Assumed risk HR(control)(per 1000)	Corresponding risk RFA(per 1000)	Quality of evidence (GRADE)
Overall survival	1-y	243(3)	95.4	92.9	1.62[0.54,4.84]	0	0.39	40.0^‡^	**907**	**940** (840 to 979)	⊕^⊖^^⊖^^⊖^ very low
	3-y	243(3)	56.2	61.9	0.80[0.48,1.34]	0	0.40	17.5†	**571**	**516** (390 to 641)	⊕^⊖^^⊖^^⊖^ very low
	5-y	243(3)	26.2	45.1	0.43[0.25,0.73]	24	0.002*	5.3†	**464**	**271** (178 to 387)	⊕^⊖^^⊖^^⊖^ very low
Disease-free survival	1-y	243(3)	55.4	69.9	0.47[0.26,0.83]	46	0.01*	6.9†	**738**	**570** (423 to 700)	⊕^⊖^^⊖^^⊖^ very low
	3-y	243(3)	13.8	30.1	0.35[0.18,0.67]	34	0.002*	6.1†	**321**	**142** (78 to 241)	⊕^⊖^^⊖^^⊖^ very low
	5-y	243(3)	2.3	12.4	0.18[0.05,0.61]	32	0.006*	9.9†	**143**	**29** (8 to 92)	⊕⊕^⊖^^⊖^ low

Ψ: The basis for the assumed risk (e.g. the median control group risk across studies) is provided in footnotes. The corresponding risk (and its 95% confidence interval) is based on the assumed risk in the comparison group and the relative effect of the intervention (and its 95%CI);

# Statistical method (M-H, random-effect model).

,NNH(Number needed to harm);

‡,NNT(Number needed to treat);

*:p<0.05

### Clinical outcome of HCC patients with tumor size smaller than 3 cm

The pooled results of RCTs showed no significant differences of the overall survival rates between groups for patients with a single HCC and tumor size ≤3 cm in diameter (level of evidence: low to moderate) (Table 4).

The pooled meta-analysis from NRCTs showed no significant differences of 1- and 3-year survival rates between groups, but the 5-year survival rate in the RFA group was lower than in the HR group (OR 0.62, 95% CI: 0.43–0.90, NNH = 4.5; p = 0.01) (level of evidence: very low). The 1-, 3- and 5-year disease-free survival rates in the RFA group were lower than in the HR group, but the 5-year disease-free survival rates for patients with a single HCC showed no significant difference between groups (OR 0.69, 95% CI: 0.35–1.36). The 1-, 3- and 5-year recurrence rates also showed no significant differences between groups, but the 5-year recurrence rate in the RFA group was lower than in the HR group for patients with Child-Pugh class A (OR 0.42, 95% CI: 0.19–0.93, NNT = 5.1) (level of evidence: moderate). The complication rate in the RFA group was lower than in the HR group (OR 0.44, 95% CI: 0.29–0.65, NNT = 11.2) (level of evidence: low), but there was no significant difference between groups for patients with Child-Pugh class A (Table 6).

**Table 6:** Summary of finding table for HCC patients with tumor size ≤3cm in NRCTs.

				Event,%		Effect estimate^#^				Illustrative comparative risks(95%CI)^Ψ^		
Indicators	Subgroup	Years	No. of Participants (studies)	RFA	HR	OR,95% CI	I^2^ (%)	P value	NNT or NNH	Assumed risk HR(control)(per 1000)	Corresponding risk RFA(per 1000)	Quality of evidence (GRADE)
Overall survival	All	1-y	2782(14)	95.1	97.2	0.72[0.50,1.04]	17	0.08	47.6†	**967**	**955** (936 to 968)	⊕^⊖^^⊖^^⊖^ **very low**
		3-y	3156(15)	80.5	86.4	0.71[0.48,1.05]^&^	68	0.09	16.9†	**811**	**753** (673 to 818)	⊕^⊖^^⊖^^⊖^ **very low**
		5-y	2636(12)	71.1	93.3	0.62[0.43,0.90]^&^	67	0.01*	4.5†	**764**	**667** (582 to 744)	⊕^⊖^^⊖^^⊖^ **very low**
	Child A	1-y	1632(9)	96.0	95.8	0.94[0.58,1.53]	22	0.81	500.0^‡^	**981**	**980** (968 to 972)	⊕⊕^⊖^^⊖^ **low**
		3-y	1632(9)	81.5	86.5	0.66[0.33,1.32]^&^	77	0.24	20.0†	**881**	**830** (710 to 907)	⊕^⊖^^⊖^^⊖^ **very low**
		5-y	1386(6)	72.4	80.9	0.64[0.38,1.08]^&^	65	0.09	11.8†	**824**	**750** (640 to 835)	⊕^⊖^^⊖^^⊖^ **very low**
	Single HCC	1-y	1516(8)	94.7	95.7	0.67[0.42,1.09]	9	0.11	100.0†	**982**	**973** (958 to 983)	⊕⊕^⊖^^⊖^ **low**
		3-y	1516(8)	80.4	87.2	0.59[0.32,1.09]^&^	70	0.09	14.7†	**898**	**839** (738 to 906)	⊕^⊖^^⊖^^⊖^ **very low**
		5-y	1516(8)	64.3	77.9	0.52[0.31,0.87]^&^	74	0.01*	7.4†	**773**	**639** (514 to 748)	⊕^⊖^^⊖^^⊖^ **very low**
	Single + Child A	1-y	1009(6)	94.6	95.2	0.83[0.48,1.46]	43	0.52	166.7†	**990**	**988** (979 to 993)	⊕⊕^⊖^^⊖^ **low**
		3-y	1009(6)	79.5	88.5	0.38[0.12,1.16]^&^	80	0.09	11.1†	**935**	**845** (633 to 943)	⊕⊕^⊖^^⊖^ **low**
		5-y	978(5)	71.3	81.7	0.58[0.28,1.19]^&^	69	0.14	9.6†	**840**	**753** (595 to 862)	⊕^⊖^^⊖^^⊖^ **very low**
Disease-free survival	All	1-y	1722(9)	71.6	85.2	0.43[0.34,0.55]	45	<0.00001*	7.4†	**854**	**716** (665 to 763)	⊕⊕^⊖^^⊖^ **low**
		3-y	1722(9)	43.1	64.3	0.45[0.31,0.64]^&^	54	<0.0001*	4.7†	**604**	**407** (321 to 494)	⊕^⊖^^⊖^^⊖^ **very low**
		5-y	1592(7)	24.8	46.2	0.50[0.26,0.97]^&^	83	0.04*	4.7†	**357**	**217** (126 to 350)	⊕⊕^⊖^^⊖^ **low**
	Child A	1-y	772(4)	76.9	86.6	0.52[0.36,0.75]	45	0.0006*	10.3†	**876**	**771** (700 to 829)	⊕⊕^⊖^^⊖^ **low**
		3-y	772(4)	57.7	71.7	0.55[0.40,0.74]	0	0.0001*	7.1†	**649**	**504** (425 to 578)	⊕⊕^⊖^^⊖^ **low**
		5-y	627(2)	42.8	53.7	0.66[0.48,0.91]	0	0.01*	9.2†	**482**	**380** (309 to 459)	⊕⊕^⊖^^⊖^ **low**
	Single HCC	1-y	1024(5)	78.7	86.4	0.54[0.39,0.75]	38	0.0003*	13.0†	**859**	**767** (704 to 820)	⊕⊕^⊖^^⊖^ **low**
		3-y	1024(5)	53.5	68.7	0.55[0.43,0.72]	0	<0.0001*	6.6†	**615**	**468** (407 to 535)	⊕⊕^⊖^^⊖^ **low**
		5-y	1024(5)	32.3	46.7	0.69[0.35,1.36]^&^	78	0.28	6.9†	**404**	**319** (192 to 480)	⊕^⊖^^⊖^^⊖^ **very low**
	Single + Child A	1-y	717(3)	75.9	87.0	0.48[0.33,0.71]	38	0.0003*	9.0†	**898**	**809** (744 to 862)	⊕⊕⊕^⊖^ **moderate**
		3-y	717(3)	79.5	88.5	0.55[0.40,0.75]	80	0.09	11.1†	**712**	**576** (497 to 650)	⊕⊕^⊖^^⊖^ **low**
		5-y	627(2)	42.8	53.7	0.66[0.48,0.91]	0	0.01*	9.2†	**482**	**380** (309 to 459)	⊕⊕^⊖^^⊖^ **low**
Recurrence-free survival	All	1-y	1467(5)	78.8	77.6	1.02[0.79,1.31]	0	0.89	83.3^‡^	**757**	**761** (711 to 803)	⊕⊕^⊖^^⊖^ **low**
		3-y	1467(5)	44.5	50.6	0.85[0.69,1.05]	17	0.13	16.4†	**493**	**453** (402 to 505)	⊕⊕^⊖^^⊖^ **low**
		5-y	1307(4)	29.2	37.4	0.76[0.40,1.43]^&^	83	0.39	12.2†	**368**	**307** (189 to 454)	⊕^⊖^^⊖^^⊖^ **very low**
	Child A	1-y	1236(4)	77.8	76.7	1.05[0.80,1.38]	0	0.71	90.9^‡^	**748**	**757** (725 to 820)	⊕⊕^⊖^^⊖^ **low**
		3-y	1236(4)	46.2	50.9	0.88[0.70,1.11]	27	0.27	21.3†	**514**	**482** (425 to 540)	⊕⊕^⊖^^⊖^ **low**
		5-y	1076(3)	32.9	38.7	0.81[0.36,1.83]^&^	89	0.61	17.2†	**370**	**322** (175 to 518)	⊕^⊖^^⊖^^⊖^ **very low**
	Single HCC	1-y	899(3)	78.2	76.0	1.03[0.74,1.42]	0	0.87	45.5	**757**	**762** (697 to 816)	⊕⊕^⊖^^⊖^ **low**
		3-y	899(3)	49.0	52.2	0.96[0.73,1.26]	26	0.79	31.3†	**521**	**511** (443 to 578)	⊕⊕^⊖^^⊖^ **low**
		5-y	899(3)	34.8	37.7	1.05[0.79,1.39]	37	0.75	34.5†	**370**	**381** (317 to 449)	⊕⊕^⊖^^⊖^ **low**
	Single + Child A	1-y	668(2)	76.0	74.3	1.09[0.76,1.56]	0	0.63	58.8^‡^	**748**	**764** (693 to 822)	⊕⊕^⊖^^⊖^ **low**
		3-y	668(2)	55.1	53.0	1.07[0.78,1.46]	0	0.68	47.6^‡^	**544**	**561** (482 to 635)	⊕^⊖^^⊖^^⊖^ **very low**
		5-y	668(2)	44.5	39.7	1.17[0.86,1.61]	0	0.32	20.8^‡^	**442**	**481** (405 to 560)	⊕^⊖^^⊖^^⊖^ **very low**
Recurrence	All	1-y	490(2)	12.9	15.4	0.83[0.49,1.40]	0	0.48	40.0^‡^	**163**	**139** (87 to 214)	⊕^⊖^^⊖^^⊖^ **very low**
		3-y	490(2)	50.7	45.2	0.95[0.29,3.11]&	87	0.94	18.1†	**493**	**480** (220 to 751)	⊕^⊖^^⊖^^⊖^ **very low**
		5-y	490(2)	66.2	59.0	0.95[0.21,4.28]^&^	91	0.94	13.9†	**638**	**626** (270 to 883)	⊕^⊖^^⊖^^⊖^ **very low**
	Child A	1-y	116(1)	18.2	18.0	1.01[0.39,2.63]	—	0.98	500.0†	**180**	**181** (79to 366)	⊕⊕^⊖^^⊖^ **low**
		3-y	116(1)	40.9	58.0	0.50[0.24,1.06]	—	0.07	5.8^‡^	**580**	**408** (249 to 594)	⊕⊕⊕^⊖^ **moderate**
		5-y	116(1)	54.5	74.0	0.42[0.19,0.93]	—	0.03*	5.1^‡^	**740**	**544** (351 to 726)	⊕⊕⊕^⊖^ **moderate**
Complication rate	All		1248(7)	7.1	16.0	0.44[0.29,0.65]	35	<0.0001*	11.2^‡^	**66**	**30** (20 to 44)	⊕⊕^⊖^^⊖^ **low**
	Child A		305(2)	11.2	31.1	0.36[0.12,1.12]^&^	55	0.08	5.0^‡^	**290**	**128** (47 to 314)	⊕^⊖^^⊖^^⊖^ **very low**

Ψ: The basis for the assumed risk (e.g. the median control group risk across studies) is provided in footnotes. The corresponding risk (and its 95% confidence interval) is based on the assumed risk in the comparison group and the relative effect of the intervention (and its 95%CI);

# Statistical method (M-H, random-effect model); & Statistical method (M-H, fixed-effect model).

,NNH(Number needed to harm);

‡,NNT(Number needed to treat);

*:p<0.05

### Clinical outcome of solitary HCC patients with tumor size smaller than 2 cm

The pooled results of three NRCTs [15], [16], [22]showed that there was no significant difference in 1-, 3- and 5-year overall survival or RFS, 3-,5-year DFS, and 1-,3-year recurrence between groups(p>0.05). However, the 1-year DFS (OR 0.22, 95%CI:0.07–0.65, NNH = 4.4; p = 0.06), 5-year recurrence(OR 0.42, 95%CI:0.19–0.93, NNT = 5.0;P = 0.03) and complication rate (OR 0.23, 95%CI:0.11–0.49, NNT = 3.1; p = 0.0001) in RFA group were lower than that of HR group(P<0.05)(level of evidence: very low)(Table 7).

**Table 7:** Summary of finding table for solitary HCC patients with tumor size ≤2 cm in NRCTs.

			Event,%		Effect estimate				Illustrative comparative risks(95%CI)^Ψ^		
Indicators	Years	No. of Participants (studies)	RFA	HR	OR,95% CI	I^2^ (%)	P value	NNT or NNH	Assumed risk HR(control)(per 1000)	Corresponding risk RFA(per 1000)	Quality of evidence (GRADE)
Overall survival	1-y	365(3)	98.4	95.5	2.60[0.73,9.29]^&^	22	0.14	34.5^‡^	981	993(974 to 998)	⊕⊕^⊖^^⊖^ **low**
	3-y	365(3)	85.7	84.6	0.65[0.10,4.01]^#^	83	0.64	90.9^‡^	920	882(535 to 979)	⊕^⊖^^⊖^^⊖^ **very low**
	5-y	365(3)	79.4	74.4	1.30[0.79,2.15]^&^	41	0.30	20.0^‡^	827	861(791 to 911)	⊕⊕^⊖^^⊖^ **low**
Disease-free survival	1-y	104(1)	67.1	89.8	0.22[0.07,0.65]^&^	—	0.006*	4.4†	904	674(397 to 860)	⊕⊕^⊖^^⊖^ **low**
	3-y	104(1)	46.4	62.1	0.54[0.25,1.17]^&^	—	0.12	6.4†	615	463(285 to 651)	⊕^⊖^^⊖^^⊖^ **very low**
	5-y	104(1)	38.0	40.7	0.92[0.42,2.03]^&^	—	0.84	37.0†	404	384(222 to 579)	⊕^⊖^^⊖^^⊖^ **very low**
Recurrence-free survival	1-y	145(1)	76.4	75.6	1.02[0.48,2.19]^&^	—	0.96	125.0^‡^	757	761(599 to 872)	⊕^⊖^^⊖^^⊖^ **very low**
	3-y	145(1)	65.2	56.1	1.40[0.72,2.74]^&^	—	0.32	11.0^‡^	568	648(486 to 783)	⊕^⊖^^⊖^^⊖^ **very low**
	5-y	145(1)	59.8	51.3	1.37[0.71,2.65]^&^	—	0.35	11.8^‡^	514	592(429 to 737)	⊕^⊖^^⊖^^⊖^ **very low**
Recurrence	1-y	116(1)	18.2	18.9	1.01[0.39,2.63]^&^	—	0.98	142.9^‡^	180	181(79 to 366)	⊕^⊖^^⊖^^⊖^ **very low**
	3-y	116(1)	40.5	57.4	0.50[0.24,1.06]^&^	—	0.07	5.9^‡^	580	408(249 to 594)	⊕⊕^⊖^^⊖^ **low**
	5-y	116(1)	54.8	74.8	0.42[0.19,0.93]^&^	—	0.03*	5.0^‡^	740	544(351 to 726)	⊕⊕^⊖^^⊖^ **low**
Complication rate		145(1)	19.1	51.4	0.23[0.11,0.49]^&^	—	0.0001*	3.1^‡^	514	196(104 to 341)	⊕⊕^⊖^^⊖^ **low**

Ψ: The basis for the assumed risk (e.g. the median control group risk across studies) is provided in footnotes. The corresponding risk (and its 95% confidence interval) is based on the assumed risk in the comparison group and the relative effect of the intervention (and its 95%CI);

# Statistical method (M-H, random-effect model);

& Statistical method (M-H, fixed-effect model).

,NNH(Number needed to harm);

‡,NNT(Number needed to treat);

*:p<0.05

### Sensitivity analysis

Among the three RCTs, the patients included by Huang et al[10] were the oldest (average age of 56 years). With exclusion of this study, the 1- and 3-year survival rates still showed no significant difference between groups, but the results of the heterogeneity test (I^2^) changed from 83–89% to 0%. The study of Feng et al[13] included more patients with cirrhosis and a high proportion (61.5% to 75%) of patients with more than two nodules. With exclusion of this study, the complication rate in the RFA group was still lower than in the HR group (RR 0.12, 95% CI: 0.06–0.24), but the results of the heterogeneity test (I^2^) changed form 74% to 0%.

### Publication bias

As is shown in Table 8, we only analyzed the publication bias for indicators included in 10 or more studies[11]. After viewing the funnel plot and Egger's test, it was found that the indicators of 5-year survival for patients with tumor size smaller than 5 cm or 3 cm in diameter showed no publication bias (P>0.05). The remaining indicators all showed that some degree of publication bias existed.

**Table 8:** Publication bias of studies which were more than ten.

Indicators	Subgroup	Years	No. of studies	Egger's test P value	Publication bias
Overall survival	HCC≤5 cm	1-y	23	<0.001	Yes
		3-y	23	<0.001	Yes
		5-y	15	0.182	No
	HCC≤5 cm and Child A	1-y	11	0.001	Yes
		3-y	12	0.006	Yes
	HCC≤3 cm	1-y	14	0.003	Yes
		3-y	15	0.005	Yes
		5-y	12	0.21	No
Disease-free survival	HCC≤5 cm	1-y	12	0.039	Yes
		3-y	11	0.011	Yes
Complication rate			15	<0.001	Yes

## Discussion

HR is considered the preferred treatment for patients meeting Milan criteria with a single nodule or multiple lesions with good liver function but unsuitable for liver transplantation [4], [5]. One prospective RCT demonstrated that the 3-year survival rate with liver resection for small hepatocellular carcinoma could reach 74.8%[13]. However, 80% of cases were unsuitable for liver resection for various reasons such as low rate of early diagnosis, poor expected function of residual liver after surgery, and anticipated serious post-operative complications [39]. Local ablation with RFA or PEI is recommended by the latest updated EASL-EORTC guidelines as the standard care for patients with Barcelona clinic liver cancer (BCLC) 0 to A level but were unsuitable for surgery in 2012[4].

RCTs of high quality and with a large sample size are considered the best sources of evidence, but RCTs are accompanied by high costs, are difficult to conduct, and often have poor external validity. RCTs performed in the field of surgery, especially with double-blinding methods, are extremely difficult. Systematic review and/or meta-analysis of RCTs are often the most feasible methods to address this situation. Previous meta-analyses worldwide have compared the long-term efficacy of RFA and HR for the treatment of small HCC, but prospective RCTs with large sample sizes were rarely included. The systematic review authors usually combined the results of RCTs and NRCTs together or mistook retrospective studies as RCTs, leading them to draw conclusions of low reliability[39]–[43], as various risks for bias or confounding factors might have existed among the study designs. However, the impact these biases on outcomes could not be ascertained because of the limited information provided in these primary studies[11]. Therefore, such results without a more strict risk assessment would inevitably lead to an erroneous estimation of effects and mislead clinical decision-making.

In this study, three RCTs without blinding were included, all with only moderate quality of evidence [10], [13], [14]. The pooled results of our meta-analysis showed no significant differences between the RFA and HR groups in overall survival and recurrence-free survival rates at 1 and 3 years and in recurrence rates at 1 year following the treatment of small hepatocellular carcinomas meeting the Milan criteria. The RFA group had higher recurrence rates at 3 and 5 years and lower complication rates when compared with the HR group. It is well-known that tumor size, number of lesions, location, liver function, the presence of portal vein invasion, the presence of vascular invasion and the width of the tumor-free margin during surgical excision were the independent prognostic factors affecting the survival of patients[44], [45]. The recurrence rate after RFA is related to incomplete tumor ablation. In case of HCC in which local curative ablative therapy was obtained by securing a safety margin[46]. It is considered that the local recurrence rate markedly differed among patients with or without a sufficient safety margin [47]. However, RFA is a minimally invasive procedure, and it is often hard to achieve a specific safety margin in three dimensions all around a large tumor[46], leading to a higher recurrence but lessened compromise of liver function, which might be one of the reasons for the relatively low postoperative complication rate. Commonly, patients with small HCC rarely dies within 1 year and the recurrence impacts the overall survival gradually. However, the 1-year survival rate of RFA in the RCTs Huang et al[10] conducted was 87%, whereas that of resection was 98%. This result is markedly different from the other two RCTs [13], [14], which might be the factors leading to the clinical heterogeneity. There are some reasons may contribute to this problem. Firstly, of 108 RFA-treated patients in the study of Huang et al[10], seventeen patients had lesions in dangerous locations, leading difficult to secure a safety margin. Secondly, the average age of included patients in both groups were much old, and they were prone to the therapy of resection. Apart form that, the rate of loss to follow-up was greater in the resection group (18/115, 15.6%) than in RFA group (7/115, 6.1%), which would probably influence the comparison between two groups.

NRCTs of high quality with large sample sizes could provide research evidence for a wider population, particularly with a greater advantage for assessing the safety of an intervention. However, NRCTs might also be more easily influenced by different kinds of bias or by unknown confounding factors[11]. In this study, twenty-five NRCTs with an average quality of “moderate” and a large pooled sample size of 11,314 subjects were included. The pooled results of the meta-analysis could have some significance for guiding clinical practice as they showed that the overall survival in the RFA group was significantly lower than in the HR group for patients with small tumor size less than 5 cm in diameter. However, identical results were suggested otherwise in respect of recurrence, complication rate, in-hospital mortality and hospital length of stay between the RCTs and NRCTs. The reason for this phenomenon was that rare RCTs with low heterogeneity and large sample size were included which showed no difference between the groups, but did not rule out the impact of potential confounding factors or bias (i.e., publication bias) in the NRCTs, leading to a possible overestimation of the survival effect in the NRCTs.

Few studies except for three retrospective ones have reported the outcome of solitary HCC with tumor size less than 2 cm. Nevertheless, the conclusion of these studies was varies. Wang JH et al[15] and Hung HH et al [22]have concluded that RFA was as effective as HR in patients with BCLC very early stage HCC, while Peng ZW et al[16] showed that percutaneous RFA was better than those of HR, especially for central HCC. In this study, we found that the overall survival and 1-, 3-year recurrence in RFA group were as equal effective as HR group, and the complication rate and 5-year recurrence in RFA group were lower than HR group. However, the overall quality of evidence evaluated by GRADE (the Grading of Recommendations Assessment, Development and Evaluation) was very low to low due to limited sample size or many known or unknown risk of bias existed in observational studies. Therefore, when applying the outcome of the evidence to clinical practice, physicians should take caution. More large-scale, well-conducted RCTs or retrospective studies focused on the topics were still needed in the further.

GRADE (2011 version) has identified five categories or factors downgrading of the quality of evidence: study limitations (risk of bias), imprecision, inconsistency, indirectness, and publication bias [48], [49]. For observational studies, the GRADE group has also identified three categories or factors raising the quality of evidence: large magnitude of effect, dose-response gradient, and plausible confounding, all of which can increase confidence in the estimated effects[50]. Therefore, our study strictly followed the quality assessment criteria of the GRADE guidelines, evaluated the quality of evidence for the important outcomes of each patient, and performed a subgroup analysis based on risk factors (i.e., the Child-Pugh class, tumor size, and number of nodules) that were likely to affect the clinical outcome, so as to reduce the impact of risk factors on clinical outcomes to some extent. It is well-known that cirrhosis is also an independent prognostic factor for patients with HCC. For example, in the study of Nishikawa H et al [20], they concluded that the presence of liver cirrhosis was the sole significant factor for recurrence-free survival. However, based on the current primary studies, it is difficult to apply a subgroup analysis for this factor individually. We suggest that more high quality RCTs or retrospective studies should be focus on this topic in the further.

### Limitations

Only three RCTs were included, and the limited sample size and high heterogeneity might affect the robustness of the clinical outcomes to some extent. Given the characteristics of the included NRCTs, we deleted some items (i.e., prospective collection of data, unbiased and blinded assessment of the study endpoint, and prospective calculation of the study size) from the MINORS score questionnaire which were unsuitable for this study, but we were convinced that it would not have an impact on the quality of evidence of the final clinical outcome.

## Conclusions

The pooled meta-analysis of the RCTs demonstrated no significant difference between groups of the overall survival, recurrence-free survival, disease-free survival and in-hospital mortality rates for early HCC with tumor size smaller than 5 cm in diameter, but the RFA group had higher recurrence rates, lower complication rates, and shorter hospital lengths of stay.

The pooled meta-analysis of the NRCTs showed no significant difference in recurrence rates between groups for patients with Child-Pugh class A or tumor size smaller than 3 cm or 3 cm to 5 cm in diameter. However, when combining the results of all patients with tumors smaller than 5 cm in diameter, it is showed that the RFA group had lower overall survival and higher recurrence rates. For patients with Child-Pugh A/B and tumor size less than 2 cm in diameter, the pooled results concluded that RFA was as effective as HR in the overall survival and 1-, 3-year recurrence, and RFA yielded lower complication rate and 5-year recurrence than HR. However, it is still need RCTs of high quality to further enhance the level of evidence.

Based on full consideration of the current available best evidence, a comprehensive conclusion can be drawn: RFA is comparable to HR with lower complication rates but with higher recurrence rates. All relevant risk factors that may affect the final outcome of patients should be considered, so as to balance minimizing recurrent HCC after RFA with improving the quality of life of patients. The authors suggest that more high quality RCTs or retrospective studies should focus on RFA versus HR for very early HCC patients and to provide the better clinical decision for physicians.

## Supporting Information

Checklist S1 (DOC)
